# Parental Antecedents of Locus of Control of Reinforcement: A Qualitative Review

**DOI:** 10.3389/fpsyg.2021.565883

**Published:** 2021-02-23

**Authors:** John S. Carton, Mikayla Ries, Stephen Nowicki

**Affiliations:** ^1^Department of Psychology, Oglethorpe University, Atlanta, GA, United States; ^2^Department of Psychology, Emory University, Atlanta, GA, United States

**Keywords:** locus of control, Rotter, antecedents, social learning theory, expectancies

## Abstract

The construct of locus of control of reinforcement has generated thousands of studies since its introduction as a psychological concept by Julian Rotter (1966). Although evidence indicates its importance for a wide range of outcomes, comparatively little research has been directed toward identification of potential developmental antecedents of internal/external expectancies. A previous review of antecedent findings (Carton and Nowicki, 1994) called for more research to be completed, particularly using observational and/or longitudinal methodologies. The current paper summarizes and evaluates antecedent research published in the intervening years since Carton and Nowicki’s review. Results largely were consistent with expectations based on Rotter’s social learning theory, although there is still a need for researchers to use observational, rather than self-report methodologies, and to include data from non-western cultures.

## Antecedents of Locus of Control of Reinforcement: A Qualitative Review

Locus of control of reinforcement [LOC] was formally introduced and defined as a psychological construct by Julian Rotter (1966), who posited that an individual with internal control expectancies will perceive events as “contingent upon his or her own relatively permanent characteristics,” in contrast to an individual with external control expectancies who “is more likely to perceive an event not to be entirely contingent upon his or her own actions but rather as a result of luck, chance, fate or unpredictable because of the forces surrounding them” (p. 2). Since Rotter’s introduction of the concept of LOC, it has become one of the most widely studied personality variables in psychology, with over 5,00,000 “hits” in Google Scholar (Nowicki and Duke, 2016).

Personality constructs such as self-efficacy (Bandura, 1992) and attribution (Abramson et al., 1978; Seligman, 1992) are sometimes used interchangeably with LOC. However, they are different constructs and should be considered separately from Rotter’s concept of a generalized expectancy. Briefly, self-efficacy is the degree to which someone believes she or he can perform a behavior (i.e., a performance expectancy), while attribution refers to judgments about causes of past outcomes (Peterson and Stunkard, 1992). Although these constructs have generated considerable research themselves, they are not equivalent to Rotter’s concept of a generalized problem-solving expectancy regarding outcomes in one’s life (i.e., an outcome expectancy). The present review will focus exclusively on studies using the generalized LOC expectancy construct as presented by Rotter (1954, 1966) within the context of his social learning theory.

Thousands of studies have investigated the relation between LOC and outcomes across a wide variety of domains. Results generally have indicated that in the presence of a range of individual differences in locus of control, more internal, rather than more external, control expectancies appear to be related to more positive and healthier outcomes (see reviews by Lefcourt, 1966, 1972, 1976, 1981, 1983, 1984; Rotter, 1966, 1975, 1982; Rotter et al., 1972; Phares, 1976; Nowicki and Duke, 2016). However, Rotter (1975) cautioned researchers not to assume internality always to be “good” and externality always to be “bad” in and of themselves, but to also consider the relation between LOC and the given psychological situation.

Unfortunately, in spite of its associations with a large range of significant outcomes, relatively few studies have attempted to identify potential parental antecedents of LOC, even though some half-century earlier Rotter (1966) highlighted the need to obtain such information. By antecedents we refer to those parental factors that precede the development of LOC and which may, through scientific investigation, be shown to have an associative or causal effect on determining one’s LOC. Nearly three decades after Rotter’s call for research identifying antecedents of LOC, Carton and Nowicki (1994) found fewer than 70 published studies on the topic. They concluded parent contingent delivery of consequences, encouragement of autonomy, and warm/supportive home environments were promising candidates. However, research to that point had relied heavily on small scale, self-report, cross-sectional, and correlational methods that limited the conclusions that could be drawn.

The goal of the current paper is to summarize the results of pertinent studies concerning parental antecedents of LOC published since Carton and Nowicki’s review and to critically evaluate whether the findings support Rotter’s theoretical assumptions concerning the development of control expectancies. We organize the antecedent literature around three themes: Parenting style, parental LOC, and parent involvement/education/homelife. Our hope is that the summarized information may not only be theoretically relevant, but also useful to those interested in developing intervention programs facilitating the growth of realistic control expectancies, that is consistent with the control available to them in the situation. We begin by briefly describing Rotter’s social learning theory and the role LOC is assumed to play in it. Then we evaluate the evidence regarding antecedents of control expectancies vis-à-vis predictions based on Rotter’s theory and, based on our evaluation, offer suggestions for future research and advice for those working with children. Consequently, our review focuses specifically on research completed with generalized LOC scales constructed to be consistent with Rotter’s concept of a generalized problem-solving expectancy.

## Rotter’s Social Learning Theory and Locus of Control of Reinforcement

Rotter’s social learning theory emphasizes the role of expectancies in determining behavior. According to Rotter (1954), behavior “is determined not only by the nature or importance of goals or reinforcements, but also by the person’s anticipation or expectancy that these goals will occur. Such expectations are determined by previous experience and can be quantified” (p. 102). As such, an expectancy “may be defined as a probability or contingency held by the subject that any specific reinforcement or group of reinforcements will occur in any given situation or situations” (p. 165). Over time, Rotter assumes such expectancies generalize across situations producing generalized expectancies, one of which is LOC.

More broadly, Rotter’s social learning theory attempts to explain behavior, or more specifically, the *potential for behavior*, with three basic variables: (1) Expectancies (E), both general and specific, which represent beliefs regarding the likelihood of a behavior causing an outcome; (2) Reinforcement Value (RV), which is the positive or negative valence of a given outcome based on learning history; and (3) The Psychological Situation, which refers to the individual’s subjective interpretation of the contextual situation. The four components can be summarized by the formula, BP = f(E & RV), which indicates Rotter’s assumption that the potential for a behavior to occur is a function of the expectation the behavior will produce the outcome and the value of the outcome for any given psychological situation (Rotter, 1954).

## Research on Antecedents of Locus of Control of Reinforcement

Having reviewed the basic components of Rotter’s theory, we move to describe and qualitatively analyze the relevant past quarter century of research concerned with identifying antecedents of his LOC construct. We begin with research investigating parenting style and children’s LOC. Please see Table 1 for a list of all studies cited in the current review organized by antecedent category.

**TABLE 1:** References by antecedent.

**Antecedent**	**References**
Parenting Style	McClun and Merrell, 1998; Khayyer, 2003; Lin and Lian, 2011; Wickline et al., 2011; Almajali, 2012; Keshavarz et al., 2013; Ahlin and Lobo Antunes, 2015
Parent LOC	Davis and Phares, 1969; Loeb, 1975; Ollendick, 1979; Chandler et al., 1980; Barling, 1982; Ackerman and Ackerman, 1989; Hoffman and Levy-Shiff, 1994; Schneewind, 1997; Tully et al., 2016; Nowicki et al., 2018b
Parent Involvement/Education/Home Life	Bryant and Trockel, 1976; Enger et al., 1994; Yates et al., 1994; Carton et al., 1996; Carton and Nowicki, 1996; Carton and Carton, 1998; Post and Robinson, 1998; Lynch et al., 2002; Khayyer, 2003; Clark et al., 2004; Cohen et al., 2008; Furnham and Cheng, 2016; Roazzi et al., 2016; Golding et al., 2017; Nowicki et al., 2018b

### Parenting Style and Children’s Locus of Control

Parents presumably play a significant role in LOC development because they are largely responsible for selecting learning experiences for their children. The development of LOC expectancies particularly appears to depend on the degree to which parents consistently and, most importantly, contingently reinforce children’s behavior-outcome sequences. As Rotter (1966) theorized, “As an infant develops and acquires more experiences, (s)he differentiates events which are causally related to preceding events and those which are not” based on learning behavior-reinforcement contingencies (p. 2).

An influential researcher of parenting, Baumrind (1991) described categories of parenting styles that have been used in several studies examining antecedents of LOC. In general, she defined parenting styles by the degree to which parents provide nurturance, but also set limits, for their children. She identified three primary styles: Authoritarian, authoritative, and permissive. Authoritarian parents are controlling and tend to use more punitive disciplinary tactics to correct their children. In contrast, authoritative parents set limits, but also display affection, support, and autonomy building practices with their children. In the third style, permissive parents emphasize affection and support, but are relatively “hands off” and de-emphasize discipline and corrective instruction in guiding their children. Results of her studies often indicated that authoritative parenting produced more positive outcomes in children than the other two styles (Baumrind, 2013), leading many LOC researchers to hypothesize that authoritative parenting might be associated with the development of internal control expectancies.

A large scale prospective longitudinal study provides data in support of the hypothesis that parenting style predicts children’s LOC. Wickline et al. (2011) used data from a national cohort study of 12,463 children born during one week in England, Scotland, and Wales and their mothers (Osborn et al., 1984). Mother-child dyads were surveyed at the time of the child’s birth and again at 5 and 10 years of age. LOC was measured by an Anglicized Children’s Nowicki-Strickland Internal External scale (Nowicki and Strickland, 1973). Analyses showed warm supportive family structures and non-authoritarian parenting styles were associated with children’s internality. In addition, a previously unidentified activity, parents reading daily to their children, was an especially significant activity at 5 years of age that predicted greater internality at age 10. To be sure of any causal linkage, LOC should have been assessed at age 5 as well as at age10, but this possible association is a candidate for future research.

Cross-sectional studies also provide support for associations between parenting style and LOC. For example, McClun and Merrell (1998) reported eighth and ninth grade children who perceived their parents as authoritative had greater internality and more positive self-concepts than those who perceived their parents as authoritarian. Similarly, Almajali (2012) found authoritative parenting was associated with greater internality, while authoritarian parenting was associated with greater externality in a group of preparatory school children in Jordan.

In sum, most research suggests that harsh, controlling parenting is associated with children’s externality and authoritatively warm, supportive parenting with children’s internality. However, some research (e.g., Keshavarz et al., 2013) from non-Western cultures has suggested the possibility that authoritarian parenting may be related to internality if children perceive it as being supportive (see also Lin and Lian, 2011). Authoritative parenting, which includes fostering autonomy in a supportive manner, appears to provide an environment conducive for a child to effectively experience behavioral contingencies, where their own actions are perceived as causally related to outcomes. In contrast, authoritarian parents may inhibit such learning because they themselves control the outcomes to a greater degree in their children’s lives. In a different fashion, permissive parents also may fail to provide an adequate environment for the learning of contingencies. Even though permissive parents are characterized as loving, their relative lack of structure, discipline, and involvement may not serve to foster the contingency learning that Rotter suggested was central to how internal expectancies develop.

### Parent Locus of Control as Antecedent to Children’s Locus of Control

Besides parenting styles, parents’ actual LOC may be a significant antecedent to children’s LOC, but this hypothesis has not received much attention. Based on the assumption that children often model their parents’ attributes, some researchers have predicted that children’s LOC will be similar to that of their parents.

However, results of most self-report, cross-sectional studies of the possible relationship between parent and child LOC have found little or no association (Davis and Phares, 1969; Loeb, 1975; Barling, 1982; Ackerman and Ackerman, 1989; Hoffman and Levy-Shiff, 1994; Morton, 1997). When an association has been found, it has been either: (1) gender related, with parent LOC being associated with daughters’, but not sons’, LOC (Ollendick, 1979) or (2) parent related, with mothers, but not fathers’, LOC being associated with children’s expectancies (Chandler et al., 1980), or (3) an interaction of both, with fewer mother-reported stressors and greater father internality correlated with daughters’, but not sons’, internality (Tully et al., 2016).

Morton (1997) offered a possible explanation for the failure of cross-sectional studies to find a significant, reliable association between parent and child LOC based on his observation that the Rotter I-E scale was used in these studies to measure parents’ LOC. Because the Rotter scale measures generalized (global) expectancies, he reasoned that a more specific scale focused uniquely on measuring parenting behavior expectancies might be more successful in tying parent LOC to child LOC. Campis et al. (1986) constructed a specific parenting LOC measure. Researchers using the scale have shown parent externality to be associated with a variety of negative children’s outcomes (e.g., Mouton and Tuma, 1988; Roberts et al., 1992). But when Morton used the specific parenting LOC test, as well as the Rotter scale, he found no association with children’s LOC as measured by the Multidimensional Measure of Children’s Perception of Control (Connell, 1985). Since no other study has used a specific parenting LOC scale and a LOC scale for children, the question of whether an association exists remains unanswered.

The lack of support for an association between parent and child LOC must be taken cautiously because most studies on this topic are limited by small sample sizes of homogeneous participants, cross-sectional designs which often excluded fathers (for exceptions, see; Ollendick, 1979; Chandler et al., 1980; Tully et al., 2016), and the administration of different locus of control tests to parents and children (with the exception of Ollendick and Tully et al.’s studies, which used parent and child forms of the Nowicki Strickland Internal External Control Scale). A more definitive answer may be found in data gathered from longitudinal studies that include a large representative population of participants and use parent and child LOC scales consistent with Rotter’s definition. Fortunately, two large scale longitudinal studies meet these criteria: Schneewind (1997) in Germany and Nowicki et al. (2018a) in England.

Schneewind’s study covered a time span of 16 years and included samples of mother-father-child triads from six different West German states (Schneewind et al., 1983). Children and their parents completed German translations of the Nowicki Strickland scales (Nowicki and Strickland, 1973; Nowicki and Duke, 1974) when the children were 12 (*n* = 285) and again 16 years later (*n* = 98). His prediction that children would model their parents’ locus of control was only supported at the later testing time and only for fathers and daughters.

Nowicki et al. (2018a) longitudinal study included an even larger and more representative population (*n* = 6,123). They analyzed data from the responses of over six thousand parents (fathers and mothers) and their children to Anglicized versions of the Adult and Child Nowicki Strickland Internal External Control Scales. The participants were part of *A Longitudinal Study of Parents and Children* (ALSPAC, Golding, 2004), an ongoing longitudinal study begun in 1991 in the city of Bristol and its environs. One unique aspect of the data set was that parents completed LOC tests *prenatally*, as well as when their children were ages 6 and 16. The children’s LOC scores also were gathered at ages 6 and 16. The findings provided some support for the modeling prediction; mothers’ and fathers’ prenatal LOC scores were related positively to those of their children’s at ages 6 and 16. Of note, no significant gender or parent differences were found among the correlations.

Thus, based on the findings from two longitudinal studies, it appears parent and child LOC may be positively associated, particularly between fathers and daughters, in young adulthood. However, the relatively modest size of the correlations means there are still additional antecedent factors to be identified besides parental LOC.

### Parental Involvement/Education/Home Life and Locus of Control

Types of parenting behaviors and the home life activities of children are considered next as possible antecedents. Nowicki et al. (2018b) investigated data gathered from mothers’ (*n* = 6,381) observations of their home environment and their children that were made before the child’s fifth birthday from within three areas: home environmental experiences, parenting, and dietary practices. For each area correlations were computed between the items completed by mothers and their children’s LOC at 6 years of age. The identified significant variables (*n* = 31) were subjected to stepwise logistic regression analysis that led to the final model of predictors (*n* = 13) of externality of children at age 6.

Support was found for the general idea that a *lack* of a warm, nurturing environment (as indicated by less breastfeeding, less cuddling at night, and less frequently being read stories) was associated with children’s externality. Children’s externality also was associated with indicators of a lack of positive parental interest in the child, as reflected by mothers of external children being more likely to have television on all day long, more frequently slapping their children, and more likely viewing pets as equally important members of the family as children.

Furthermore, children’s externality was associated with more parental attention to washing hands before eating and a greater likelihood of children eating a diet of processed food. These findings may suggest parents of externals are more attentive to the physical, rather than emotional, needs of the child; an idea that requires exploration in future research.

Another important aspect of parental involvement is the degree to which parents may “enable” their children. Lynch et al. (2002) found too much or too little parental involvement was associated with children’s externality. Too much involvement, to the extent parents actually control most outcomes for their children, may teach children outcomes are not the result of their own behavior but, rather, due to the efforts of others. Too little involvement, perhaps to a neglectful degree, may deprive children of the support they need to examine behavior-outcome sequences and learn how to cope with failure, both basic to learning appropriate internality. These findings are consistent with those described earlier involving parenting styles.

One reason why parents may differ in how they treat their children is the amount of education they have obtained. Furnham and Cheng (2016) reviewed the relevant literature and determined a correlation exists between higher levels of parent education and greater internality in children. Their conclusion is supported by a more recent, large-scale prospective study (Golding et al., 2017). The authors suggested more educated parents may produce a greater number of stable organized learning experiences for their children compared to those offered by their less educated peers.

The identification of parenting behaviors associated with children’s LOC also has been gathered from studies in which parent-child interactions were observed directly. Carton et al. (1996) predicted that parents of children with internal control expectancies would provide more contingent reinforcement, support, and encouragement of autonomy than parents of children with external control expectancies. In their study, mothers and their second-grade children were videotaped while interacting on a series of puzzles, including a difficult one that elicited maternal involvement. The results indicated that, for boys, those with internal control expectancies had mothers who offered more contingent support (e.g., suggestions for how their children might solve the difficult puzzle), but were less likely to intrude or take over the puzzle for them. In contrast, boys with external control expectancies were more likely to have mothers who contingently ignored their struggles and/or intervened by completing the puzzles for them. These findings are consistent with those reported earlier indicating an association between lack of parental emotional support and children’s externality (Nowicki et al., 2018b).

Carton and Nowicki (1996) conducted another observational study examining parenting behaviors and home environment for LOC development in children. The authors videotaped 7- and 8-year old children and their mothers’ interactions while the children worked on several tasks. They also asked mothers to complete measures of their general home environment and stressful life events. Analyses revealed that, compared to children with external control expectancies, those with internal expectancies experienced fewer stressful events, less maternal control, and more maternal warmth.

In a third observational study of elementary aged children, Carton and Carton (1998) found greater maternal warmth, as defined by mothers’ nonverbal behaviors (e.g., frequency of smiles, positive touches, and time gazing) was associated with children’s internality. Consistent with this finding, Enger et al. (1994) found that, the higher the internality, the greater likelihood children had of receiving positive parental responses, but in this study in the form of *verbal* communication. Thus, internal LOC expectancies appear to be associated with parental warmth, whether communicated nonverbally or verbally.

The results of several studies suggest that stressful childhood environments may be associated with children’s externality. The findings are consistent with Rotter’s assumptions and with Bryant and Trockel (1976) explanation of how different kinds of stressful environments may affect LOC development. As the latter noted, “…to the extent that individuals particularly try to make sense of their stressful or perceived unusual life experiences, it is conceivable that variables such as critical stressful life events are also related to one’s (external) locus of control orientation” (Bryant and Trockel, 1976, p. 266). Particular stressors already linked to children’s externality include situations where children grow up with parental alcoholism (Post and Robinson, 1998), excessive physical punishment (Khayyer, 2003), psychiatric difficulties (Yates et al., 1994), maltreatment (Roazzi et al., 2016), intellectual deficits (Clark et al., 2004), or physical disorders such as Cerebral Palsy (Cohen et al., 2008).

In summary, support exists for an association between internality and greater parental education, warmth, support, and encouragement of autonomy based on findings from cross-sectional and longitudinal studies; a conclusion consistent with the one offered by Carton and Nowicki, 1994 in their review. However, since their review, results from studies in non-Western countries and certain ethnic groups within Western cultures suggest the antecedent-LOC relation may be more complex than previously thought. For example, there may be cultural differences in how support is demonstrated by parents and valued by children. We note that Rotter’s original formula for predicting behavior includes reinforcement value and situational factors, as well as expectancies. It would seem logical that those additional constructs might be applicable when investigating the development of control expectancies and, to the best of our knowledge, few if any studies on antecedents have included them.

## Recommendations for Future Research

### Self-Report vs. Observational Designs

Although Carton and Nowicki’s original 1994 critical review called for more studies using observational methodologies to investigate the development of control expectancies, most researchers continue to rely primarily upon self-report approaches. While self-report data are relatively easier to obtain than those acquired through other methodologies, their scientific usefulness is limited for several reasons. First, self-report methodologies involving adult participants reflecting on parenting they received years ago as young children are problematic because respondents’ memories may not be accurate. Second, self-report surveys often fail to ask about specific contingent parenting behaviors, making it difficult to accurately evaluate the results vis-a-vis Rotter’s predictions about expectancy development.

Carton and Nowicki (1994) suggested two ways of reducing the potential bias in self-report methodology: (1) sampling younger participants and/or (2) asking parents to answer information about their own parenting behaviors. Both suggestions were applied in some studies, providing important corroborating evidence for predictions based on Rotter’s theory (e.g., Keshavarz et al., 2013; Ahlin and Lobo Antunes, 2015; Tully et al., 2016; Nowicki et al., 2018a).

Only three studies have utilized observational methodologies to investigate parenting behaviors and children’s locus of control orientation since Carton and Nowicki’s review (Carton and Nowicki, 1996; Carton et al., 1996; Carton and Carton, 1998). The results of all three provided evidence that largely corroborated self-report data and supported predictions based on Rotter’s theory. An advantage of such studies is that they provide specific behavioral exemplars for parents interested in promoting the growth of appropriate internal LOC expectancies in their children.

### Consistency vs. Contingency

Besides noting methodological shortcomings, Carton and Nowicki called for greater awareness of what Rotter meant by “contingency learning” and how it differs from the concept of consistency. Contingency refers to when the occurrence of one event is dependent on the prior occurrence of another event. A contingent consequence can be formulated as an “if-then” statement: If you achieve X, then Y will happen; if you do not achieve X, then Y will not happen. In the case of children’s control expectancies, Rotter assumed parental reinforcement contingent on children’s actions would be associated with the development of internal control expectancies. Conversely, consequences administered by parents that are not contingent on the children’s behavior would be associated with the development of external control expectancies.

Note that consistency refers simply to the reliability of the behavior or outcome. A parent could consistently act in a non-contingent fashion toward their child, thus scoring high on consistency but low on contingency. In the only two studies on LOC antecedents that accurately measured parental contingent behaviors, results supported Rotter’s presumed association between contingent outcomes and children’s internality (Skinner, 1986; Carton et al., 1996).

### Focus on Parents vs. Others

Most LOC antecedent studies have concentrated on parents and their children. Regrettably, little research has been done to evaluate the potential role of teachers or other significant adults outside of the home. This is true despite the fact that “children between the ages of five and eighteen spend most of their waking hours either in school or at home working on assignments given to them in school. American school children spend more time in school than do most other children in the world” (Nowicki, 2016, p. 89). Yet the possible effect of teachers, coaches, and other adults on control expectancies is largely unknown. Interestingly, some colleges now offer noncredit classes in which the goal is to teach students how their own behavior plays an important role in their personal and academic difficulties and successes based on the premise that LOC can be influenced by people other than parents (Downing and Brennan, 2019).

### Gender Differences

Many of the antecedent studies have not systematically gathered information concerning how gender may affect the learning of internal and external control expectancies. In general, when scales constructed to be consistent with Rotter’s definition are used, average scores for males and females do not differ (e.g.,Kulas, 1996; Wickline et al., 2011; Almajali, 2012). It is important to note that, although gender differences in LOC scores are infrequent, the predictive validity of LOC expectancies may differ by gender in some cases. One example is the prediction of academic achievement, where LOC more accurately predicts male, than female, performance outcomes (e. g., Kalechstein and Nowicki, 1997). Another example is from Schneewind (1997), in which father-daughter, but not father-son, LOC scores were significantly related, even though average LOC scores between sons and daughters did not differ.

While most studies have found LOC scores do not differ by gender, there are exceptions. For example, Gursoy and Bicakci (2007) found males to be more internal than females using a sample of Turkish lower socioeconomic children. Consistent with research noted earlier in this review regarding the potential mediating effects of cultural variables, the authors noted that Turkish females have more limited behavioral and social opportunities than males, which may impede their chances to learn from a wider range of behavioral contingencies.

### Measuring Locus of Control as Defined by Rotter

When Rotter (1966) introduced LOC as a psychological construct, he also presented a self-report questionnaire constructed to be consistent with his definition; a questionnaire frequently used to assess adults’ LOC since then. However, as time passed, the very popularity of the LOC concept created problems. As Nowicki and Duke (2016) concluded, “What once was a clearly defined global generalized expectancy construct that functioned as a major component of Rotter’s social learning theory (1954), (LOC) appears to have morphed into a complex array of concepts that sometimes appear to be only tangentially related to the original LOC-R concept introduced by Rotter” (p. 150). Decades ago, Skinner (1996) found hundreds of definitions being offered for “locus of control” and a like number of tests being used to allegedly measure the construct. Unfortunately, some researchers failed to reference Rotter’s social learning theory and the definition of LOC it offered. It should be noted that Rotter emphasized the concept of “expectancy” and gave it a major place in his theory, but often this concept and even the word “expectancy” are lacking in descriptions of many tests purporting to measure LOC or in studies with titles suggesting they are investigating LOC as defined by Rotter.

Skinner (1996) conclusion about the myriad of LOC definitions and scales remains relevant today: “Even a cursory consideration of the area reveals a large number of terms which, although different, nevertheless seem to be interrelated and partially overlapping” (p. 549). She went on to state that, “Within the total set of terms, some appear to be different labels for the same construct” and “probably most confusing are cases in which the same term is used to refer to different constructs” (p. 550). This jumble of terms and tests makes it difficult to generalize results across studies and to determine if the results are relevant to Rotter’s theory.

To add to the confusion are the many content specific LOC tests, such as academic achievement (Crandall et al., 1965), health (Wallston et al., 1978), work (Spector, 1988), safety (Wuebker, 1986) and even God (Wallston et al., 1999), as well as tests attempting to assess different sources of externality (e. g., Levenson, 1975). Some of the tests have gathered considerable construct validity evidence, but many have not, and few have provided support of their test’s “incremental” validity; that is, the ability of their specific LOC test to predict outcomes consistently and significantly better than generalized expectancy measures. Consequently, we do not know, for the most part, if specific content LOC expectancy tests are identifying new sources of variance or gathering the same information as the generalized LOC questionnaires.

To help clarify the measurement issues germane to the identification of developmental antecedents of LOC, we suggest the following. First, researchers use Rotter’s definition of LOC as a generalized problem-solving expectancy. Second, they administer tests constructed to be consistent with Rotter’s definition accompanied by evidence of construct validity supporting their effectiveness. Third, investigators should note when they are departing from Rotter’s definition and the tests constructed to be consistent with it. Fourth, when introducing tests of “locus of control” researchers include information showing how they are related to generalized LOC measures, evidence of their incremental validity, and findings supportive of the tests’ use with the study population. With such information, researchers can more accurately distinguish evidence of antecedents for Rotter’s defined LOC construct from other constructs.

## Conclusion

We acknowledge the majority of our findings are based on data from Western English speaking populations and further studies need to be completed to determine their broader external validity. It also is important to note that childhood is not the only time-period in which LOC expectancies can be modified. Rotter (1954, 1966) social learning theory and subsequent empirical data indicate that certain salient events can modify LOC at other points in development (e.g., Nowicki et al., 2018a,c). That being said, Rotter’s theory suggests that childhood is when the largest changes tend to occur in the development of generalized expectancies.

While studies showing the importance of the LOC construct as defined by Rotter (1966) continue to be published at an impressively high rate, research focused on identifying antecedents of internal and external generalized expectancies lags far behind. The results of the present qualitative review are somewhat consistent with those of Carton and Nowicki (1994). Parenting that allows children the freedom to experience the outcomes of their behavior, accompanied by the communication of warmth, support, and feedback when children fail, continues to be critical in the development of appropriate internality. However, researchers have not yet investigated the possible impact on children’s LOC of other significant adults in children’s lives, such as teachers and coaches.

Identifying relevant antecedents of LOC requires the use of appropriate tests and the application of Rotter’s LOC definition and social learning theory. It is more obvious now than it was at the time of Carton and Nowicki (1994) review that confusion exists about what is being called “locus of control” and how it is being measured. We urge researchers to be clear in describing the LOC test they use and the LOC definition they apply.

We hope that raising awareness of LOC and summarizing the existing antecedent research will lead to more focused investigations of this important topic. LOC is one of the most researched and highly cited constructs in the history of psychology, in part because it has been shown to be a significant predictor of a diverse range of outcomes. If confirmed by results of studies from various cultures, the trend toward greater externality is troubling. As Rotter (1971) warned so many years ago in words that may be appropriate today: “*Our society has so many critical problems that it desperately needs as many active, participating internal-minded members as possible. If feelings of external control, alienation and powerlessness continue to grow, we may be heading for a society of dropouts – each person sitting back, watching the world go by” (p. 59).*

## Author Contributions

All authors listed have made a substantial, direct and intellectual contribution to the work, and approved it for publication.

## Conflict of Interest

The authors declare that the research was conducted in the absence of any commercial or financial relationships that could be construed as a potential conflict of interest.
